# Nicotinic Mitigation of Neuroinflammation and Oxidative Stress After Chronic Sleep Deprivation

**DOI:** 10.3389/fimmu.2019.02546

**Published:** 2019-10-29

**Authors:** Rong Xue, Yahui Wan, Xiaoqian Sun, Xuan Zhang, Wei Gao, Wei Wu

**Affiliations:** ^1^Department of Neurology, Tianjin Medical University General Hospital, Tianjin, China; ^2^Department of Neurology, Tianjin Medical University General Hospital Airport Hospital, Tianjin, China

**Keywords:** chronic sleep deprivation, inflammation, glial cells, α7-nAChR, oxidative stress

## Abstract

Sleep deprivation negatively influences all aspects of health. Oxidative stress and inflammatory responses induced by sleep deprivation participate in its adverse effects but the regulatory mechanisms to counteract them remain poorly understood. In mice subjected to sleep deprivation for 7 days, we found activation of microglia and astrocyte accompanied by down-regulation of α7 nicotinic acetylcholine receptor (α7-nAChR) and reduced activation of downstream PI3K/AKT/GSK-3β. These changes occurred with an increase of pro-inflammatory factors, together with reduced levels of anti-inflammatory factors, transcriptor Nrf-2, and anti-oxidant enzyme HO-1. Administration of an α7-nAChR agonist PHA-543613 induced activation of PI3K/AKT/GSK-3β, and reversed changes in pro-inflammatory and anti-inflammatory factors, Nrf-2 and HO-1. These results suggest that stimulation of α7-nAChR reduce neuroinflammation and oxidative stress after chronic sleep deprivation.

## Introduction

Sleep deprivation triggers an array of inflammatory responses (1). In the CNS, emerging evidence suggests that sleep deprivation activates astrocytes and microglia, leading to increased levels of pro-inflammatory factors and neural injury (2, 3). In addition, sleep deprivation represents an oxidative challenge to the brain. Although both neuroinflammation and oxidative stress are key contributors to adverse effects of sleep deprivation, little is known about the counter-regulatory mechanisms to mitigate these factors.

The α7 nicotinic acetylcholine receptors (α7-nAChR) is a crucial player in the regulation of immune responses and oxidative stress in the CNS and periphery (4). More than a traditional ligand-gated ion channel, α7-nAChR is involved in the processes of learning, memory consolidation, movement, and attention (5, 6). Stimulation of α7-nAChR is linked to the activation of multiple intracellular signaling pathways (7–9). In the downstream of α7-nAChR, activation of PI3K/AKT inhibits the glycogen synthase kinase-3β (GSK-3β) (10, 11), which induces the expression of transcriptor Nrf-2 and antioxidant enzyme HO-1 and inhibits the release of pro-inflammatory cytokines (12–14). However, the role of α7-nAChR and its downstream signaling pathway remain elusive in the context of sleep deprivation.

In periphery, macrophages are the inflammatory cells, while α7-nAChR on the macrophages plays a critical role in the inflammatory response. Activation of macrophages can produce a variety of inflammatory factors such as TNF-α, IL-1β, IL-6, and HMGB etc., which may penetrate into the central nervous system through the damaged blood-brain barrier and cause neuroinflammation (15, 16). Increasing evidence revealed that systemic inflammation can influence the progression of several diseases like multiple sclerosis (MS), Alzheimer's disease (AD), Parkinson's disease (PD), and postoperative cognitive dysfunction (POCD) (17–19). A cohort clinical study showed that 5 nights of sleep restriction increased the production of proinflammatory cytokines including IL-1β, IL-6, and IL-17 in peripheral blood, it indicating that sleep deprivation may cause systemic inflammation (20). None the while in the sleep deprivation model, whether inflammatory cytokines released in peripheral blood influence the central nervous system through the blood-brain barrier remains unclear.

In this study, we investigated the influences of sleep deprivation for 7 days on the expression α7-nAChR and the activity of its downstream PI3K/AKT/GSK-3β pathway in neuroglia in mice hippocampus and examined the effects of a selective α7-nAChR agonist PHA-543613 on sleep deprivation-induced inflammation and oxidative stress. In addition, we analyzed the systemic inflammatory response and the change of the blood-brain barrier induced by sleep deprivation, to investigate the effects of systemic inflammation on neuroinflammation.

## Materials and Methods

### Animals

This study was carried out in accordance with the NIH Guide for the Care and Use of Laboratory Animals and the Ethics Committee of Animal Experiments of Tianjin Medical University. The protocol was approved by the Ethics Committee of Animal Experiments of Tianjin Medical University. Male C57BL/6 mice, 8–10 weeks old, were used. The mice were randomly assigned into each experimental group. All mice were housed in pathogen-free conditions of the vivarium facilities and all surgeries were performed with animals under anesthesia. Reporting of this study complies with the Animal Research: Reporting *in vivo* Experiments (ARRIVE) guidelines.

### Sleep Deprivation

Sleep deprivation was achieved using modified multiple platforms method as our previous study (21, 22). The water box is characterized as 50 cm in length, a width of 30 cm, and a height 30 cm. Eight rounded platforms were evenly placed in the water box, with a diameter of 3 cm, a height of 6 cm, and an interval between the platforms of 7 cm. Only six mice were in the containers at a time, so that they could move without restriction. The water level was controlled to just below the platform for a depth of 1 cm and was maintained at a temperature of 22 ± 2°C. Enough food and water were placed in the top of the box, so the mice could get to it without difficulty.

Before sleep deprivation, mice were placed in the modified multiple platform water box for 2 h every day, for 3 consecutive days to adapt to the environment. Sleep deprivation began at 9:00 a.m., after which the mice were taken out of the deprivation boxes from 17:00 to 21:00 every day, and placed in cages and given free access to food and water for 4 h. Sleep deprivation lasted for a total of 7 days, and the water in the box was replaced every day.

### Experimental Groups and Drug Administration

Animals were divided into three groups randomly: Cage control Group (CC group); chronic sleep deprivation for 7 days (SD group); intraperitoneal (i.p.) administration of α7-nAChR agonist PHA-543613 after chronic sleep deprivation for 7 days (SD + PHA-543613 group). The circadian system regulates many physiological functions including inflammatory responses. Plenty of researches proved that circadian clocks persist in immune cells including microglia (23–25), and clock genes are involved in regulating immunological activities. Thus, animals were fed under standard illumination parameters (12-h light/dark cycle) to avoid the influence of different circadian rhythms on the neuroinflammatory response of microglia and astrocytes.

PHA-543613 (Sigma-Aldrich), a potent, high-affinity and selective a7-nAChR agonist, it is characterized by rapid brain penetration (26). Based on previous studies (26–28), PHA-543613 was administrated at 6 mg/kg by intraperitoneal injection immediately 6 h after chronic sleep deprivation, and continued for 3 consecutive days until the experiment terminated. The saline vehicle was administrated at 6 mg/kg by intraperitoneal injection in the CC group. All animals were operated on at 9 a.m.

### Behavioral Testing

Spatial learning and memory was assessed by morris water maze (MWM) test (29). Briefly, the experimental apparatus consisted of a round water tank (150 cm wide and 50 cm high) filled with water (25°C) and surrounded by visual cues around the tank. An invisible platform (15 cm wide and 35 cm high) was placed 1 cm below the surface of the water. The spatial learning and memory ability of mice were evaluated by the number of times of platform, time in target quadrant, and average swimming speed. Data collection was automated by a video image motion analyzer.

Mice were tested in different quadrants four times a day. In each trial, the mice were randomly released into the water from one of the four quadrants with their face toward the wall of the maze. The location of the platform remained fixed during the acquisition phase and the rats were allowed to swim for 60 s to find the invisible platform. After the animal found the platform, it was allowed to remain there for 20–30 s and then returned to the cage to wait another 20–30 s before the start of the next trial. The time spent to find the invisible platform were calculated and analyzed. The mice trained at 8:30 a.m. daily after sleep deprivation. After the fourth trials, the animals were kept warm for an hour and then put back in a sleep deprivation box. After sleep deprivation of 7 days, a probe phase was performed to assess spatial memory retention. In the probe test, the platform was removed and each mouse was allowed to swim for 60 s. The time and distance spent in the target quadrant and the number of crossing in the target quadrant were analyzed as a criterion for spatial memory retention. In this test, the ability of animals to escape latency to a visible platform was evaluated.

### Immunofluorescence and Cell Counting

After behavioral testing, the mice were anesthetized with chloral hydrate (30 mg/kg, i.p.) and transaortically perfused with cold PBS. The brains were removed and postfixed in 4% paraformaldehyde overnight at 4°C. Then brains were dehydrated through 15 and 30% sucrose. After successful dehydration, brains were embedded in OCT. Immunofluorescence was performed on 8-mm frozen sections of the hippocampus. After the slides were brought to room temperature for 30 min, the tissue sections were fixed in 4% paraformaldehyde for 15 min. The tissue sections were permeabilized in 0.3% Triton X 100 for 30 min and then blocked with 5% bovine serum albumin (Sigma-Aldrich) for 30 min at 37°C and the sections were exposed to the primary antibodies overnight at 4°C. The next day they were washed in PBS and then incubated for 60 min at room temperature with species-appropriate fluorochrome-conjugated secondary antibodies. The primary antibodies used were goat anti-Iba-1 (1:500; Abcam, Cambridge, United Kingdom), rabbit anti-GFAP (1:1,000, Abcam, Cambridge, United Kingdom), rat anti α7-nAChR (1:100, Santa Cruz Biotechnology), rabbit anti- Albumin (1:100, ABclonal, China). The mean number of cells stained positive was calculated from 3 randomly selected microscopic fields, both in the penumbra of each section, and 3 consecutive sections were analyzed for each brain section. Data are expressed as mean number of cells per visual field, counted under high magnification (3200).

### Real-Time RT-PCR

Total RNA was extracted from hippocampal tissues with Trizol. The concentration of RNA was quantified by ultraviolet spectrophotometry at 260/280 nm. Total RNA was reverse-transcribed into complementary DNA (cDNA) by using the SuperScript First-Strand Synthesis System for RT-PCR (Invitrogen). The primers used to measure gene expression are the following TNF-α (F: CAAGGGACAAGGCTGCCCCG; R: GCAGGGGCTCTTGACGGCAG), IL-1β (F: TCCAGGATGAGGACATGAGCAC; R: GAACG CACACACCAGCAGGTTA), IFN-γ (F: AGCTCTTCCTCATGGCTGTT; R: TTTGCCAGTTCCTCCAGATA), MCP-1 (F: ACGCTTCTGGGCCTGTTGTT; R: CCTGCTGCTGGTGATTCTCT), Arg-1 (F: TTAGGCCAAGGTGCTTGCTGCC; R: TACCATGGCCCTGAGGAGGTTC), CD206 (F: CAAGGAAGGTTGGCATTTGT; R: CCTTTCAGTCCTTTGCAAGC), TGF-β (F: TGCGCTTGCAGACATTAAAA; R: CGTCAAAAGACAGCCACTCA), YM-1 (F: CGAGGTAATGAGTGGGTTGG; R: CACGGCACCTCCTAAATTGT), α7-nAChR (F: AACCATGCGCCGTAGGACA; R: CTCAGCCACAAGCAGCATGAA), GAPDH (F: GCCAAGGCTGTGGGCAAGGT; R: TCTCCAGGCGGCACGCAGA) (F = forward, R = reverse). All the procedures were strictly performed as per instructions. The PCR programe was run at the following cycling conditions: 44 cycles of 10 s at 95°C, 30 s at 58°C, and 20 s at 72°C Specificity of the PCR product was confirmed by examination of dissociation reaction plots. A distinct single peak indicated that a single DNA sequence was amplified during PCR. The expression levels of the mRNAs were analyzed by the method of 2–ΔΔCt. The result was calculated as levels of target mRNAs relative to GAPDH.

### Western Blot

Hippocampus tissue was removed from each group and was homogenized by sonication in ristocetin-induced platelet aggregation buffer containing protease and phosphatase inhibitors (Complete Protease Inhibitor Cocktail and PhosStop Phosphatase Inhibitor Cocktail; both from Roche Diagnostics). Protein concentration was assessed with BCA Assay Kit (Solarbio Life Science, Beijing, China), and 10–20 μg protein was loaded per lane. Equal amounts proteins were separated on 10% Tris-glycine gradient gels (Bio-Rad) at 80 V and the voltage was then raised to 120 V. The proteins were transferred onto PVDF membranes for 1.5 h at 4°C with a current of 100 V. The membranes were blocked for 2 h at room temperature in 5% non-fat dry milk powder dissolved in buffer, incubated with primary antibody overnight at 4°C. The primary antibodies used were rat anti-α7-nAChR (1:200 Santa Cruz Biotechnology)rabbit anti- p-AKT(1:1,000, Cell Signal Technology, America), rabbit anti-AKT (1:1,000, Cell Signal Technology, America), rabbit anti-p-GSK3β (1:1,000, Cell Signal technology, America), rabbit anti-GSK (1:1,000, Cell Signal technology, America), rabbit anti-Nrf2 (1:2,000, Abcam, Cambridge, United Kingdom), rabbit anti-HO-1 (1:1,000, Abcam, Cambridge, United Kingdom), mouse anti-GAPDH (1:2,000, TransGen Biotech Co., Beijing, China), mouse anti-β-action (1:5,000, TransGen Biotech Co., Beijing, China). Next day, the membranes were washed 3 times for 10 min each at room temperature, incubated with a horseradish peroxidase–coupled secondary antibody (1:5,000; TransGen Biotech Co., Beijing, China) for 1 h, and washed 3 times for 10 min each at room temperature. The membrane was scanned on an Odyssey Infrared Imaging System (Bio-Rad). The optical densities of target protein bands were measured and normalized to the corresponding b-actin bands. Samples were run in triplicate or quadruplicate.

### ELISA for Cytokine Profile

Blood was collected by cardiac puncture under deep isoflurane anesthesia and then centrifuged at 2,000 g for 10 min at 4°C. Plasma cytokines were stored at 80°C for further analysis. Levels of TNF-α, IL-1β, IL-6 were measured using commercially available enzyme-linked immunosorbent assay (ELISA) kits from Abcam (Cambridge, United Kingdom). The sensitivities of the assays were <9.1 pg/ml for TNF-α, <1 pg/ml for IL-1β, <11.3 pg/ml for IL-6.

### Statistical Analysis

Data were presented as mean ± SEM. One-way analysis of variance (ANOVA) with *post-hoc* tests to compare among the three groups. Data with a non-parametric distribution were analyzed with the Mann–Whitney test. A *P* < 0.05 was considered significant. SPSS for Windows version 17.0 software (SPSS, Inc., Chicago, IL, USA) was used for analysis.

## Results

### Reduced Expression of α7-nAChR in Mouse Hippocampus After Chronic Sleep Deprivation

**Figure 1:**
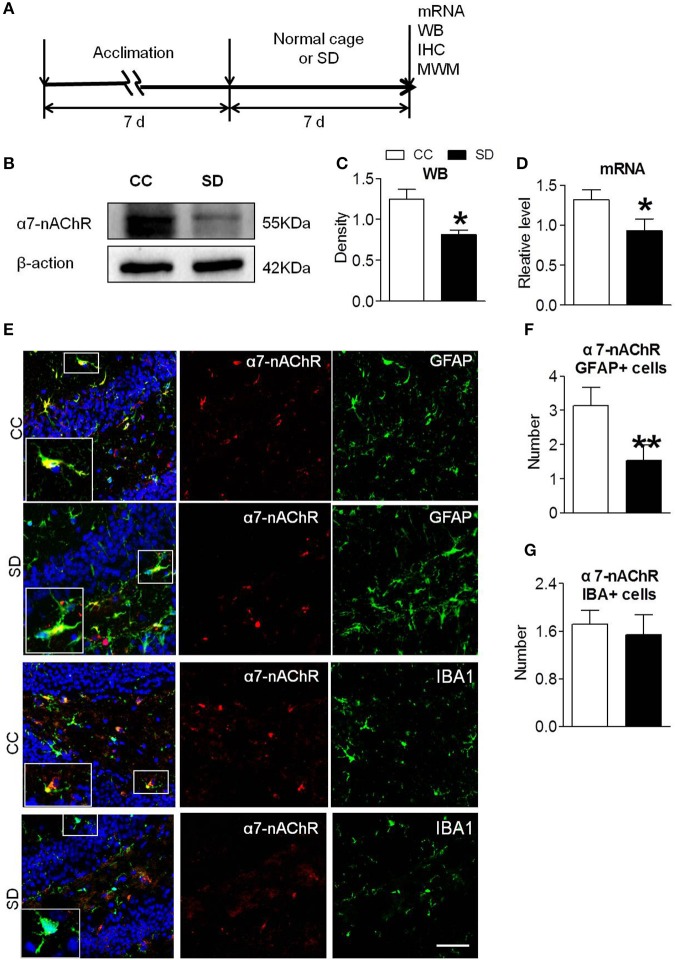
Down-regulated α7-nAChR expression in mice hippocampus after chronic sleep deprivation. **(A)** Schematic diagram depicting the experimental procedures. Before sleep deprivation, the mice in SD group was adapted to the environment in the modified multiple platforms for 7 days, 2 h per day. All mice were housed with 12 h light/dark cycles during the experiment and were operated on after sleep deprivation at 9 a.m. **(B,C)** Western blots of α7-nAChR in hippocampus, compared to control group, chronic sleep deprivation for 7 days significantly decreased expression of protein α7-nAChR in hippocampus (*P < 0.05). **(D)** Chronic sleep deprivation for 7 days significantly decreased mRNA expression of α7-nAChR in hippocampus. **(E–G)** Immunofluorescence staining of α7-nAChR and quantitation of α7-nAChR with glail cells. Less α7-nAChR/GFAP+ and α7-nAChR/IBA+ cells were observed in SD group compared with CC group. The scale bar represents 50 mm. The results are expressed as mean ± SEM,*P < 0.05, **P < 0.01, vs. control group. CC, Cage control; SD, sleep deprivation.

Sleep deprivation was achieved using modified multiple platforms method (Figure 1A). Western blot and RT-PCR were used to determine the expression of α7-nAChR after chronic sleep deprivation for 7 days. As shown in Figures 1B,C, the protein expressions of α7-nAChR after sleep deprivation were decreased compared to the control group in the hippocampus. The mRNA expressions of α7-nAChR after sleep deprivation were decreased compared to the control group in the hippocampus (Figure 1D). Immunofluorescence analysis revealed that α7-nAChR expression was significantly down-regulated in glial cells after sleep deprivation (Figure 1E). α7-nAChRs expressing of GFAP+ astrocyte cell after chronic sleep deprivation was also decreased (Figure 1F), but α7-nAChR s expressing of IBA+ microglia cells was not significantly different between the both groups (Figure 1G).

### Suppressed PI3K/AKT/GSK-3β Pathway in Mouse Hippocampus After Chronic Sleep Deprivation

**Figure 2:**
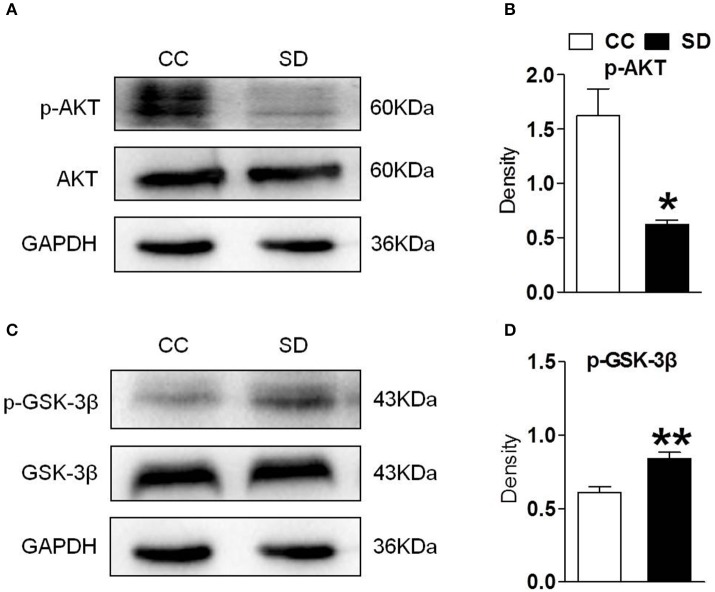
Hippocampus PI3K/AKT/GSK-3β was suppressed after chronic sleep deprivation. Western blot was used to examined the protein p-AKT, AKT, p-GSK-3β, GSK-3β. **(A,B)** Chronic sleep deprivation for 7 days significantly decreased expression of p-AKT in hippocampus. **(C,D)** Chronic sleep deprivation for 7 days increased the expression of p-GSK-3βin hippocampus. The results are expressed as mean ± SEM,*P < 0.05, **P < 0.01, vs. control group.

PI3K/AKT/GSK-3β is an important downstream pathway of α7-nAChR in the hippocampus. To assess whether α7-nAChR regulate PI3K/AKT/GSK-3β after chronic sleep deprivation, we examined the expression of p-AKT, p-GSK-3β after chronic sleep deprivation with Western blot. Sleep deprivation for 7 days induced decreased expression of protein p-AKT and increased expression of protein p-GSK-3β in the hippocampus. But the expression of AKT and GSK-3β had no change (Figures 2A–D). Above results suggested chronic sleep deprivation for 7 days inhibited PI3K/AKT/GSK-3β pathway.

### Stimulation of α7-nAChR Induced Activation of PI3K/AKT/GSK-3β Pathway

**Figure 3:**
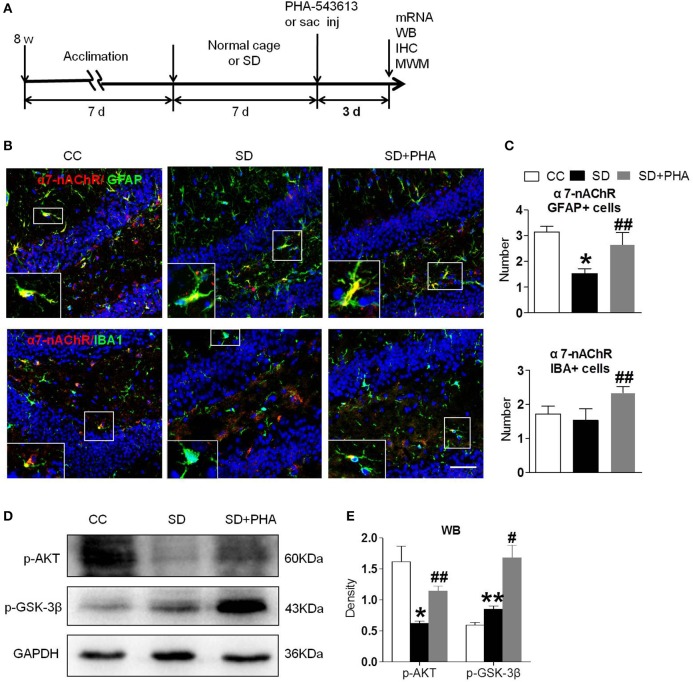
Agonist of α7-nAChR up-regulated expression of α7-nAChR on glial cells and activated PI3K/AKT/GSK-3β in hippocampus. **(A)** Schematic diagram depicting the experimental procedures. Before sleep deprivation, the mice in SD group was adapted to the environment in the modified multiple platforms for 7 days, 2 h per day. After sleep deprivation 7 days, PHA-543613 or salin was administrated at 6 mg/kg by intraperitoneal injection immediately 6 h after chronic sleep deprivation, and it was given 3 consecutive days until the experiment ended. All mice were housed with 12 h light/dark cycles during the experiment and were operated on after sleep deprivation at 9 a.m. **(B,C)** Immunofluorescent staining show counts of α7-nAChR/GFAP+ cells and α7-nAChR/IBA+ cells in the hippocampus after PHA-543613 treatment in chronic sleep deprivation. After administration of PHA-543613 for 3 consecutive days, a significant increase in α7-nAChR/GFAP+ cells was observed, the α7-nAChR/IBA+ cells were also increased, but it had no significant difference. **(D,E)** Western blot show the expression of p-AKT, p-GSK-3β. The expression of p-AKT, p-GSK-3β were increased after PHA-543613 treatment for 3 consecutive days. Data are expressed as means ± SEM,*P < 0.05, **P < 0.01, vs. control group, ^#^*P* < 0.05, ^##^*P* < 0.01, vs. SD + PHA-543613 group. CC, Cage control; SD, sleep deprivation; SD + PHA, intraperitoneal (i.p.) administration of α7-nAChR agonist PHA-543613 (6 mg/kg) after chronic sleep deprivation for 7 days.

To assess whether the α7-nAChR agonist PHA-543613 affect the expression of the PI3K/AKT/GSK-3β in hippocampus, PHA-543613 (6 mg/kg) or vehicle (9% NaCl) was injected intraperitoneally for 3 consecutive days starting 6 h later after chronic sleep deprivation (Figure 3A). PHA-543613 up regulated the expression of α7-nAChR on glial cells (Figures 3B,C), and increased the expression of protein p-AKT, p-GSK-3β (Figures 3D,E).

### Stimulation of α7-nAChR Alleviated Reduced Neuroinflammation and Oxidative Stress After Chronic Sleep Deprivation

**Figure 4:**
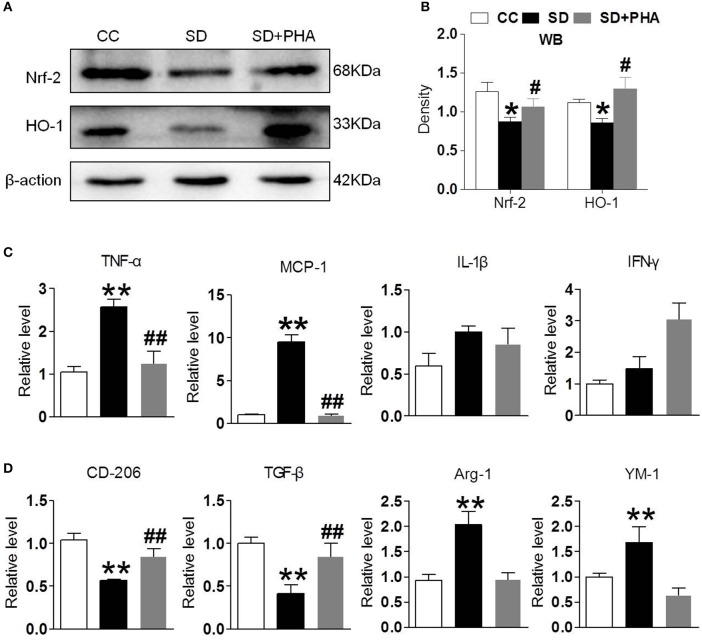
Activated α7-nAChR-PI3K/AKT/GSK-3β alleviates inflammatory response and oxygen stress induced from chronic sleep deprivation. **(A,B)** Western blot showed expression of Nrf-2, HO-1 in the hippocampus after PHA-543613 treatment in chronic sleep deprivation. Chronic sleep deprivation inhibited the expression of Nrf-2, HO-1 in the hippocampus and PHA-543613 action was just the reverse. **(C,D)** Real time PCR was used to reveal the production of proinflammatory and anti-inflammatory cytokines. After chronic sleep deprivation, the releases of proinflammatory cytokine were increased and the anti-inflammatory cytokine were decreased. However, after PHA-543613 treatment, the release of pro-inflammatory was decreased and the anti-inflammatory cytokines was increased. Data are expressed as means ± SEM.*P < 0.05, **P < 0.01, vs. control group, ^#^*P* < 0.05, ^##^*P* < 0.01, vs. SD + PHA-543613 group.

Nrf-2 and HO-1 are the major anti-oxidant enzymes and play an important role in antioxidant stress. Sleep deprivation activates glial cells, which release proinflammatory and anti-inflammatory cytokines. To assess whether α7-nAChR regulate the anti-oxidant system and inflammatory response via PI3K/AKT/GSK-3β, the expression of Nrf-2, HO-1, and inflammatory, anti-inflammatory cytokines were also assessed. We found a significant decrease in the expression of Nrf-2, HO-1 after chronic sleep deprivation. However, after PHA-543613 treatment, the expression of Nrf-2, HO-1 increased (Figures 4A,B). In addition, chronic sleep deprivation promoted the release of pro-inflammatory cytokines and inhibited release of anti-inflammatory cytokines. As Figures 4C,D showed, PHA-543613 attenuated the release of TNF-α, MCP-1 and promoted the release of CD206, TGF-β. However, we did not find a decrease of IL-1β, IFN-γ, and an increase of Arg-1, YM-1, which we think may be related to the fact that, some glial cells are still unactivated although PHA-543613 has been treated for the SD mice. This result suggests that α7-nAChR regulate anti-oxidant system and inflammatory response via PI3K/AKT/GSK-3β pathway in chronic sleep deprivation, and activating α7-nAChR via PHA-543613 reduces inflammatory cytokines and promotes antioxidant enzyme in some degree.

### Sleep Deprivation Mediates Peripheral Inflammatory, BBB Disruption, and No More Macrophages Infiltration in the Hippocampus

**Figure 5:**
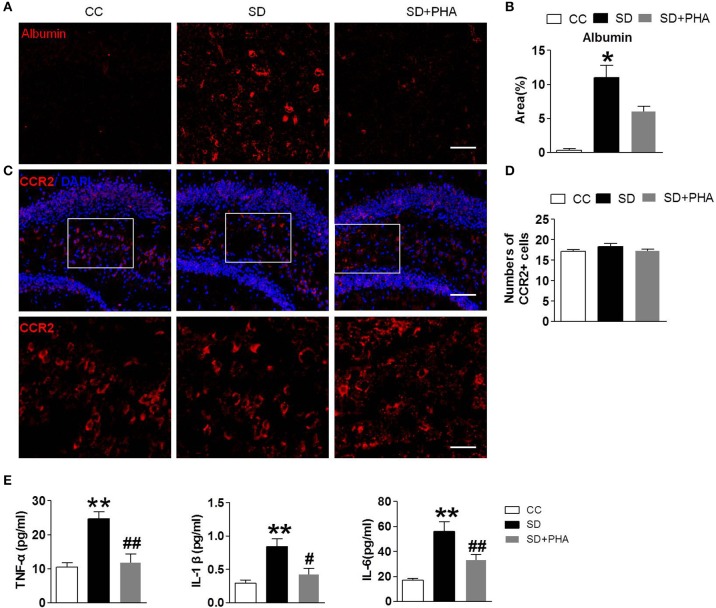
Sleep deprivation mediates peripheral inflammatory, BBB disruption and no more macrophages infiltration in hippocampus. Imfluorescence micrographs of brain cryosections showing the distribution of albumin and macrophages in the hippocampus of mice. Red labeling represents albumin extravasation as a marker of BBB damage and the CCR2 as a marker of macrophages. **(A,B)** Compared to control group, sleep deprivation increased the expression of albumin in the hippocampus. After PHA-543613 treatment, the albumin deposition in hippocampus was decreased compared to the sleep deprivation. **(C,D)** Conversely, there were no more macrophages in the hippocampus after sleep deprivation than control group. **(E)** The release of peripheral inflammatory factor TNF-α, IL-1β, IL-6 was increased after sleep deprivation, activation of the α-nAChR by PHA-543613 reduced the release of TNF-α, IL-1β, IL-6. Data are expressed as means ± SEM. *P < 0.05, **P < 0.01, vs. control group, ^#^*P* < 0.05, ^##^*P* < 0.01, vs. SD + PHA-543613 group.

In periphery, macrophages are inflammatory cells, α7-nAChR on the macrophages plays a critical role in the inflammatory response. We selectively assessed the integrity of the BBB, system inflammation and the macrophages following sleep deprivation. We assessed the integrity of the BBB by measuring albumin deposition after sleep deprivation. BBB disruption did not occur under physiological conditions (see Figures 5A,B). Following chronic sleep deprivation 7 days, there was significant albumin deposition confined to the hippocampus. PHA-543613 treatment reduced the accumulation of albumin in the hippocampus (Figures 5A,B). To investigate the effect of peripheral inflammatory response on hippocampus after sleep deprivation, ELISA and immunofluorescence labeling was applied. As shown in Figures 5C,D, there was no significance in the numbers of macrophages among three groups, but the release of peripheral inflammatory factor TNF-α, IL-1, IL-6 was increased, activation of the α-nAChR by PHA-543613 can alleviate the systemic inflammation (Figure 5E).

### PHA-543613 Treatment Prevented Cognitive Decline After Sleep Deprivation

**Figure 6:**
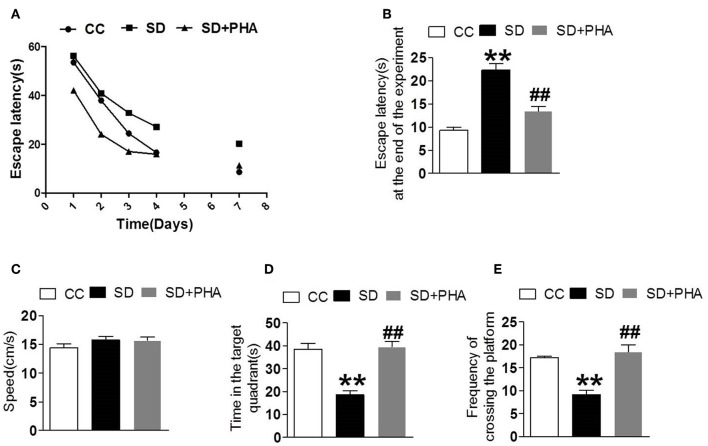
Effects of cholinergic modulation on memory function following sleep deprivation. Water morris maze was used to test the learning and memory function of mice. **(A,B)** The escape latency of mice in the three groups decreased gradually and compared with CC group, the escape latency significant increased in SD group, the PHA-543613 treatment reduced the escape latency at the end of experiment. **(C–E)** Compared with the CC group, sleep deprivation decreased the frequency of crossing the platform, and shorten the time in the target quadrant. Following the PHA-543613 treatment, the frequency of crossing platforms and the time in the target quadrant was prolonged compared to the SD group. There was no significance in swimming speed among the groups. Data are expressed as means ± SEM. *P < 0.05, **P < 0.01, vs. control group, ^#^*P* < 0.05, ^##^*P* < 0.01, vs. SD + PHA-543613 group.

We explored the effects of cholinergic modulation on cognitive decline assessing hippocampal-dependent memory function using the morris water maze and noted that sleep deprivation caused memory impairment. The escape latency, the time in the target quadrant and the frequency of crossing platforms were longer than the control group. Conversely, PHA-543613 treatment ameliorated memory impairment (Figure 6).

## Discussion

This study provides novel evidence that stimulation of α7-nAChR alleviates neuroinflammation, oxidative stress and cognitive decline after chronic sleep deprivation. As documented here, sleep deprivation for 7 days induced downregulation of α7-nAChR in microglia and astrocytes, together with the inhibited PI3K/AKT/GSK-3β pathway. PHA-543613 treatment induced the upregulation of α7-nAChR and activation of PI3K/AKT/GSK-3β. The hippocampal inflammation, oxidative stress, and behavioral responses was attenuated following PHA-543613 treatment. These results suggest α7-nAChR as a relevant biomarker in the hippocampus for inflammation and oxidative stress after chronic sleep deprivation. Stimulation of α7-nAChR may provide benefit against adverse effects induced by sleep deprivation.

In the central nervous system, α7-nAChR is mainly distributed in neurons (30, 31). More and more studies have found the expression of α7-nAChR on the surfaces of astrocytes (32) and microglia (33, 34), T cells (35), B cells (36), and monocytes (37, 38). Hippocampal cells are dominated by neurons, while microglia and astrocytes are main immune inflammatory cells in the brain, which have multiple capabilities including antigen presentation and production of pro-inflammatory or anti-inflammatory factors, etc. (39–41). Chronic sleep deprivation activates microglia and astrocyte (3, 42), so microglia and astrocytes can be potential targets to restrict brain inflammation and oxidative stress after sleep deprivation.

α7-nAChR-dependent pathways are critically involved in several CNS disorders, including schizophrenia, depression, anxiety, stroke, Alzheimer's disease, and Parkinson disease (43, 44), and increasingly studies highlight the importance of a cholinergic reflex in resolving the inflammatory pathogenesis of several diseases including rheumatoid arthritis (20) and colitis (45). Stimulation of the α7 nicotinic acetylcholine receptor protects against neuroinflammation and improved hippocampal dependent memory dysfunction, through the modulation of NF-κB activation in monocytes and regulation of the oxidative stress response through NADPH signaling (46). However, how α7-nAChR changes and takes effect after sleep deprivation in central nervous system remains unknown.

In our study, we found that α7-nAChR occurred in both microglia and astrocytes. After chronic sleep deprivation, the expression of α7-nAChR in microglia and astrocytes were reduced, and the pro-inflammatory factors were increased, while the anti-inflammatory factors and antioxidant enzymes were reduced. α7-nAChR agonist PHA-543613 can alter microglia and astrocytes response and attenuates inflammatory injury and enhances anti-inflammatory factors and antioxidant enzymes. This results support our hypothesis that targeting α7-nAChR may provide protection against sleep deprivation. To understand the possible mechanisms by which α7-nAChR might regulate inflammatory response and oxidative stress, we further examined the state of PI3K/AKT/GSK-3β signaling pathway. The results demonstrate that chronic sleep deprivation inhibited PI3K/AKT/GSK-3β signaling pathway which can be reversed by α7-nAChR agonist PHA-543613. Therefore, α7-nAChR may inhibit inflammation and oxidative stress via the modulation of microglia and astrocyte α7-nAChR/PI3K/AKT/GSK-3β pathway.

Sleep deprivation induces the production of proinflammatory cytokines in peripheral blood, which could act on peripheral macrophages to activate NF-KB or Toll-like receptors, enhancing the release of pro-inflammatory cytokines like TNF-α, HMGB, IL-6. TNF-α can compromise endothelial function and permeabilize the BBB (15). In this study, we found that the systemic TNF-α and IL-1β, IL-6 were increased and the BBB disrupt following sleep deprivation. Macrophages though, was slightly found in the brain, which was conflict with some studies, and need to be confirmed with living cell imaging in the future.

Cholinergic signaling, in particularα7 nAChR–dependent PI3K/AKT/GSK-3β pathway are critically involved in several disorders, including schizophrenia, stroke, Alzheimer's disease, myocardial ischemia reperfusion and experimental autoimmune encephalomyelitis and can provide protection against inflammatory injury (27, 47–52). We showed that PHA-543613 treatment significantly reduced the production of pro-inflammatory factors, increased the release of anti-inflammatory factors and antioxidant enzymes after chronic sleep deprivation. The morris water maze data demonstrated worsening of cognition after chronic sleep deprivation, while PHA-543613 treatment prevented the cognitive decline, therefore raising the possibility that cholinergic dysfunction is a risk factor for the development of cognitive decline after chronic sleep deprivation by interfering with resolution of the neuroinflammatory response.

Our study also showed that PHA-543613 also significantly increased the expression of antioxidant enzymes Nrf-2 and HO-1 after chronic sleep deprivation. Nrf-2 and HO-1 are the main antioxidant enzymes, which can inhibit oxidative stress. Animal experiments have proved that both of Nrf-2 and HO-1 have anti-inflammatory effects, inhibiting the release of inflammatory factors such as TNF-α, IL-1β, and MIP-1, and playing an important role in resisting oxidative stress (53–55). Whether the increased expression of Nrf-2 and HO-1 after chronic sleep deprivation is further involved in inhibiting the release of inflammatory factors and promoting the release of anti-inflammatory factors remains to be further studied.

In summary, this is the first study to report the role of α7-nAChR in the modulation of inflammatory response and oxidative stress in chronic sleep deprivation model. Our data reveal that α7-nAChR attenuates cognitive decline and neuroinflammation and oxidative stress through PI3/AKT/GSK-3β pathway. The findings broaden the neuroinflammatory mechanisms in sleep deprivation and are conducive to the therapy of brain injury aroused from sleep deprivation.

## Data Availability Statement

All datasets generated for this study are included in the article/supplementary material.

## Ethics Statement

This study was carried out in accordance with the NIH Guide for the Care and Use of Laboratory Animals and the Ethics Committee of Animal Experiments of Tianjin Medical University. The protocol was approved by the Ethics Committee of Animal Experiments of Tianjin Medical University.

## Author Contributions

WW, RX, and YW conceived and designed the study. RX and YW designed and performed the experiments, analyzed, and interpreted data. WW, RX, and YW wrote and revised the manuscript. XS, XZ, and WG participated in the data acquisition, analysis, and interpretation. All authors had critically revised and approved the final version of the manuscript.

### Conflict of Interest

The authors declare that the research was conducted in the absence of any commercial or financial relationships that could be construed as a potential conflict of interest.

## References

[1] Van Leeuwen WM Lehto M Karisola P Lindholm H Luukkonen R Sallinen M. Sleep restriction increases the risk of developing cardiovascular diseases by augmenting proinflammatory responses through IL-17 and CRP. PLoS ONE. (2009) 4:e4589. 10.1371/journal.pone.0004589 19240794 PMC2643002

[2] Chennaoui M Gomez-Merino D Drogou C Geoffroy H Dispersyn G Langrume C. Effects of exercise on brain and peripheral inflammatory biomarkers induced by total sleep deprivation in rats. J Inflamm. (2015) 12:56. 10.1186/s12950-015-0102-3 26425116 PMC4588685

[3] Bellesi M de Vivo L Chini M Gilli F Tononi G Cirelli C. Sleep Loss promotes astrocytic phagocytosis and microglial activation in mouse cerebral cortex. J Neurosci. (2017) 37:5263–73. 10.1523/JNEUROSCI.3981-16.2017 28539349 PMC5456108

[4] Ren C Tong YL Li JC Lu ZQ Yao YM. The protective effect of alpha 7 nicotinic acetylcholine receptor activation on critical illness and its mechanism. Int J Biol Sci. (2017) 13:46–56. 10.7150/ijbs.16404 28123345 PMC5264260

[5] Fucile S Renzi M Lax P Eusebi F. Fractional Ca(2+) current through human neuronal alpha7 nicotinic acetylcholine receptors. Cell Calcium. (2003) 34:205–9. 10.1016/S0143-4160(03)00071-X 12810063

[6] Park HJ Lee PH Ahn YW Choi YJ Lee G Lee DY. Neuroprotective effect of nicotine on dopaminergic neurons by anti-inflammatory action. Eur J Neurosci. (2007) 26:79–89. 10.1111/j.1460-9568.2007.05636.x 17581257

[7] Wang H Liao H Ochani M Justiniani M Lin X Yang L. Cholinergic agonists inhibit HMGB1 release and improve survival in experimental sepsis. Nat Med. (2004) 10:1216–21. 10.1038/nm1124 15502843

[8] Yue Y Liu R Cheng W Hu Y Li J Pan X. GTS-21 attenuates lipopolysaccharide-induced inflammatory cytokine production *in vitro* by modulating the Akt and NF-κB signaling pathway through the α7 nicotinic acetylcholine receptor. Int Immunopharmacol. (2015) 29:504–12. 10.1016/j.intimp.2015.10.005 26490221

[9] Cui WY Zhao S Polanowska-Grabowska R Wang J Wei J Dash B. Identification and characterization of poly(I:C)-induced molecular responses attenuated by nicotine in mouse macrophages. Mol Pharmacol. (2013) 83:61–72. 10.1124/mol.112.081497 23028093 PMC3533466

[10] Borovikova LV Ivanova S Zhang M Yang H Botchkina GI Watkins LR. Vagus nerve stimulation attenuates the systemic inflammatory response to endotoxin. Nature. (2000) 405:458–62. 10.1038/35013070 10839541

[11] Parrish WR Gallowitsch-Puerta M Czura CJ Tracey KJ. Experimental therapeutic strategies for severe sepsis: mediators and mechanisms. Ann N Y Acad Sci. (2008) 1144:210–36. 10.1196/annals.1418.011 19076379

[12] Charpantier E Wiesner A Huh KH Ogier R Hoda JC Allaman G. Alpha7 neuronal nicotinic acetylcholine receptors are negatively regulated by tyrosine phosphorylation and Src-family kinases. J Neurosci. (2005) 25:9836–49. 10.1523/JNEUROSCI.3497-05.2005 16251431 PMC6725579

[13] McCubrey JA Steelman LS Bertrand FE Davis NM Sokolosky M Abrams SL. GSK-3 as potential target for therapeutic intervention in cancer. Oncotarget. (2014) 5:2881–911. 10.18632/oncotarget.2037 24931005 PMC4102778

[14] Brugge J Hung MC Mills GB. A new mutational AKTivation in the PI3K pathway. Cancer Cell. (2007) 12:104–7. 10.1016/j.ccr.2007.07.014 17692802

[15] Terrando N Eriksson LI Kyu Ryu J Yang T Monaco C Feldmann M. Resolving postoperative neuroinflammation and cognitive decline. Ann Neurol. (2011) 70:986–95. 10.1002/ana.22664 22190370 PMC4556354

[16] He HJ Wang Y Le Y Duan KM Yan XB Liao Q. Surgery upregulates high mobility group box-1 and disrupts the blood-brain barrier causing cognitive dysfunction in aged rats. CNS Neurosci Therap. (2012) 18:994–1002. 10.1111/cns.12018 23078219 PMC6493557

[17] Gyoneva S Davalos D Biswas D Swanger SA Garnier-Amblard E Loth F. Systemic inflammation regulates microglial responses to tissue damage *in vivo*. Glia. (2014) 62:1345–60. 10.1002/glia.22686 24807189 PMC4408916

[18] Murta V Ferrari CC. Influence of peripheral inflammation on the progression of multiple sclerosis: evidence from the clinic and experimental animal models. Mol Cell Neurosci. (2013) 53:6–13. 10.1016/j.mcn.2012.06.004 22771835

[19] Han Z Li L Wang L Degos V Maze M Su H. Alpha-7 nicotinic acetylcholine receptor agonist treatment reduces neuroinflammation, oxidative stress, and brain injury in mice with ischemic stroke and bone fracture. J Neurochem. (2014) 131:498–508. 10.1111/jnc.12817 25040630 PMC4221541

[20] van Maanen MA Lebre MC van der Poll T LaRosa GJ Elbaum D Vervoordeldonk MJ. Stimulation of nicotinic acetylcholine receptors attenuates collagen-induced arthritis in mice. Arthritis Rheum. (2009) 60:114–22. 10.1002/art.24177 19116908

[21] Cui L Xue R Zhang X Chen S Wan Y Wu W. Sleep deprivation inhibits proliferation of adult hippocampal neural progenitor cells by a mechanism involving IL-17 and p38 MAPK. Brain Res. (2019) 1714:81–7. 10.1016/j.brainres.2019.01.024 30677408

[22] Guzman-Marin R Suntsova N Bashir T Nienhuis R Szymusiak R McGinty D. Rapid eye movement sleep deprivation contributes to reduction of neurogenesis in the hippocampal dentate gyrus of the adult rat. Sleep. (2008) 31:167–75. 10.1093/sleep/31.2.167 18274263 PMC2225569

[23] Musiek ES Lim MM Yang G Bauer AQ Qi L Lee Y. Circadian clock proteins regulate neuronal redox homeostasis and neurodegeneration. J Clin Invest. (2013) 123:5389–400. 10.1172/JCI70317 24270424 PMC3859381

[24] Fonken LK Frank MG Kitt MM Barrientos RM Watkins LR Maier SF. Microglia inflammatory responses are controlled by an intrinsic circadian clock. Brain Behav Immun. (2015) 45:171–9. 10.1016/j.bbi.2014.11.009 25433170 PMC4386638

[25] Fonken LK Kitt MM Gaudet AD Barrientos RM Watkins LR Maier SF. Diminished circadian rhythms in hippocampal microglia may contribute to age-related neuroinflammatory sensitization. Neurobiol Aging. (2016) 47:102–12. 10.1016/j.neurobiolaging.2016.07.019 27568094 PMC5813798

[26] Wishka DG Walker DP Yates KM Reitz SC Jia S Myers JK Discovery of N-[(3R)-1-azabicyclo[2.2.2]oct-3-yl]furo[2,3-c]pyridine-5-carboxamide, an agonist of the alpha7 nicotinic acetylcholine receptor, for the potential treatment of cognitive deficits in schizophrenia: synthesis and structure–activity relationship. J Med Chem. (2006) 49:4425–36. 10.1021/jm0602413 16821801

[27] Krafft PR Caner B Klebe D Rolland WB Tang J Zhang JH. PHA-543613 preserves blood-brain barrier integrity after intracerebral hemorrhage in mice. Stroke. (2013) 44:1743–7. 10.1161/STROKEAHA.111.000427 23613493 PMC3696522

[28] Bali ZK Inkeller J Csurgyók R Bruszt N Horváth H Hernádi I. Differential effects of α7 nicotinic receptor agonist PHA-543613 on spatial memory performance of rats in two distinct pharmacological dementia models. Behav Brain Res. (2015) 278:404–10. 10.1016/j.bbr.2014.10.030 25447295

[29] Nezhadi A Sheibani V Esmaeilpour K Shabani M Esmaeili-Mahani S. Neurosteroid allopregnanolone attenuates cognitive dysfunctions in 6-OHDA-induced rat model of Parkinson's disease. Behav Brain Res. (2016) 305:258–64. 10.1016/j.bbr.2016.03.019 26970579

[30] Hurst R Rollema H Bertrand D. Nicotinic acetylcholine receptors: from basic science to therapeutics. Pharmacol Ther. (2013) 137:22–54. 10.1016/j.pharmthera.2012.08.012 22925690

[31] Dineley KT Pandya AA Yakel JL. Nicotinic ACh receptors as therapeutic targets in CNS disorders. Trends Pharmacol Sci. (2015) 36:96–108. 10.1016/j.tips.2014.12.002 25639674 PMC4324614

[32] Shen JX Yakel JL. Functional α7 nicotinic ACh receptors on astrocytes in rat hippocampal CA1 slices. J Mol Neurosci. (2012) 48:14–21. 10.1007/s12031-012-9719-3 22351110 PMC3530828

[33] Shytle RD Mori T Townsend K Vendrame M Sun N Zeng J. Cholinergic modulation of microglial activation by alpha 7 nicotinic receptors. J Neurochem. (2004) 89:337–43. 10.1046/j.1471-4159.2004.02347.x 15056277

[34] De Simone R Ajmone-Cat MA Carnevale D Minghetti L. Activation of alpha7 nicotinic acetylcholine receptor by nicotine selectively up-regulates cyclooxygenase-2 and prostaglandin E2 in rat microglial cultures. J Neuroinflammation. (2005) 2:4. 10.1186/1742-2094-2-4 15670336 PMC548670

[35] Razani-Boroujerdi S Boyd RT Davila-Garcia MI Nandi JS Mishra NC Singh SP. T cells express alpha7-nicotinic acetylcholine receptor subunits that require a functional TCR and leukocyte-specific protein tyrosine kinase for nicotine-induced Ca2+ response. J Immunol. (2007) 179:2889–98. 10.4049/jimmunol.179.5.2889 17709503

[36] Skok MV Kalashnik EN Koval LN Tsetlin VI Utkin YN Changeux JP. Functional nicotinic acetylcholine receptors are expressed in B lymphocyte-derived cell lines. Mol Pharmacol. (2003) 64:885–9. 10.1124/mol.64.4.885 14500745

[37] Hamano R Takahashi HK Iwagaki H Yoshino T Nishibori M Tanaka N. Stimulation of α7 nicotinic acetylcholine receptor inhibits CD14 and the toll-like receptor 4 expression in human monocytes. Shock. (2006) 26:358–64. 10.1097/01.shk.0000228168.86845.60 16980882

[38] Yoshikawa H Kurokawa M Ozaki N Nara K Atou K Takada E. Nicotine inhibits the production of proinflammatory mediators in human monocytes by suppression of I-kappaB phosphorylation and nuclear factor-kappaB transcriptional activity through nicotinic acetylcholine receptor alpha7. Clin Exp Immunol. (2006) 146:116–23. 10.1111/j.1365-2249.2006.03169.x 16968406 PMC1809735

[39] Tay TL Savage JC Hui CW Bisht K Tremblay MÈ. Microglia across the lifespan: from origin to function in brain development, plasticity and cognition. J Physiol. (2017) 595:1929–45. 10.1113/JP272134 27104646 PMC5350449

[40] Brown GC Neher JJ. Microglial phagocytosis of live neurons. Nat Rev Neurosci. (2014). 15:209–16. 10.1038/nrn3710 24646669

[41] Chung WS Allen NJ Eroglu C. Astrocytes control synapse formation, function, and elimination. Cold Spring Harb Perspect Biol. (2015) 7:a020370. 10.1101/cshperspect.a020370 25663667 PMC4527946

[42] Calcia MA Bonsall DR Bloomfield PS Selvaraj S Barichello T Howes OD. Stress and neuroinflammation: a systematic review of the effects of stress on microglia and the implications for mental illness. Psychopharmacology. (2016) 233:1637–50. 10.1007/s00213-016-4218-9 26847047 PMC4828495

[43] Taly A Corringer PJ Guedin D Lestage P Changeux JP. Nicotinic receptors: allosteric transitions and therapeutic targets in the nervous system. Nat Rev Drug Discov. (2009) 8:733–50. 10.1038/nrd2927 19721446

[44] Han Z Shen F He Y Degos V Camus M Maze M. Activation of α-7 nicotinic acetylcholine receptor reduces ischemic stroke injury through reduction of pro-inflammatory macrophages and oxidative stress. PLoS ONE. (2014) 9:e105711. 10.1371/journal.pone.0105711 25157794 PMC4144901

[45] Ghia JE Blennerhassett P El-Sharkawy RT Collins SM. The protective effect of the vagus nerve in a murine model of chronic relapsing colitis. Am J Physiol Gastrointest Liver Physiol. (2007) 293:G711–8. 10.1152/ajpgi.00240.2007 17673544

[46] Terrando N Yang T Ryu JK Newton PT Monaco C Feldmann M. Stimulation of the α7 nicotinic acetylcholine receptor protects against neuroinflammation after tibia fracture and endotoxemia in mice. Mol Med. (2014) 20:667–75. 10.2119/molmed.2014.00143 25365546 PMC4398668

[47] Krafft PR Altay O Rolland WB Duris K Lekic T Tang J α7 nicotinic acetylcholine receptor agonism confers neuroprotection through GSK-3β inhibition in a mouse model of intracerebral hemorrhage. Stroke. (2012) 243:844–50. 10.1161/STROKEAHA.111.639989 PMC3293395 22207510

[48] Mavropoulos SA Khan NS Levy ACJ Faliks BT Sison CP Pavlov VA. Nicotinic acetylcholine receptor-mediated protection of the rat heart exposed to ischemia reperfusion. Mol Med. (2017) 23:120–33. 10.2119/molmed.2017.00091 28598489 PMC5522950

[49] Nizri E Hamra-Amitay Y Sicsic C Lavon I Brenner T. Anti-inflammatory properties of cholinergic up-regulation: a new role for acetylcholinesterase inhibitors. Neuropharmacology. (2006) 50:540–7. 10.1016/j.neuropharm.2005.10.013 16336980

[50] Liu Y Zeng X Hui Y Zhu C Wu J Taylor DH. Activation of α7 nicotinic acetylcholine receptors protects astrocytes against oxidative stress-induced apoptosis: implications for Parkinson's disease. Neuropharmacology. (2015) 91:87–96. 10.1016/j.neuropharm.2014.11.028 25486621

[51] Egea J Buendia I Parada E Navarro E León R Lopez MG. Anti-inflammatory role of microglialα7-nAChRs and its role in neuroprotection. Biochem Pharmacol. (2015) 97:463–72. 10.1016/j.bcp.2015.07.032 26232730

[52] Munro G Hansen R Erichsen H Timmermann D Christensen J Hansen H. The α7 nicotinic ACh receptor agonist compound B and positive allosteric modulator PNU-120596 both alleviate inflammatory hyperalgesia and cytokine release in the rat. Br J Pharmacol. (2012) 167:421–35. 10.1111/j.1476-5381.2012.02003.x 22536953 PMC3481048

[53] Huang Y Li W Su ZY Kong AN. The complexity of the Nrf2 pathway: beyond the antioxidant response. J Nutr Biochem. (2015) 26:1401–13. 10.1016/j.jnutbio.2015.08.001 26419687 PMC4785809

[54] Zhang DD. Mechanistic studies of the Nrf2-Keap1 signaling pathway. Drug Metab Rev. (2006) 38:769–89. 10.1080/03602530600971974 17145701

[55] Kim J Cha YN Surh YJ. A protective role of nuclear factor-erythroid 2-related factor-2 (Nrf2) in inflammatory disorders. Mutat Res. (2010) 690:12–23. 10.1016/j.mrfmmm.2009.09.007 19799917

